# Electrowinning for Room-Temperature Ironmaking: Mapping the Electrochemical Aqueous Iron Interface

**DOI:** 10.1021/acs.jpcc.4c01867

**Published:** 2024-08-22

**Authors:** Lance Kavalsky, Venkatasubramanian Viswanathan

**Affiliations:** † Department of Mechanical Engineering, Carnegie Mellon University, Pittsburgh, Pennsylvania 15213, United States; ‡ Department of Mechanical Engineering, University of Michigan, Ann Arbor, Michigan 48109, United States; ¶ Department of Aerospace Engineering, University of Michigan, Ann Arbor, Michigan 48109, United States

## Abstract

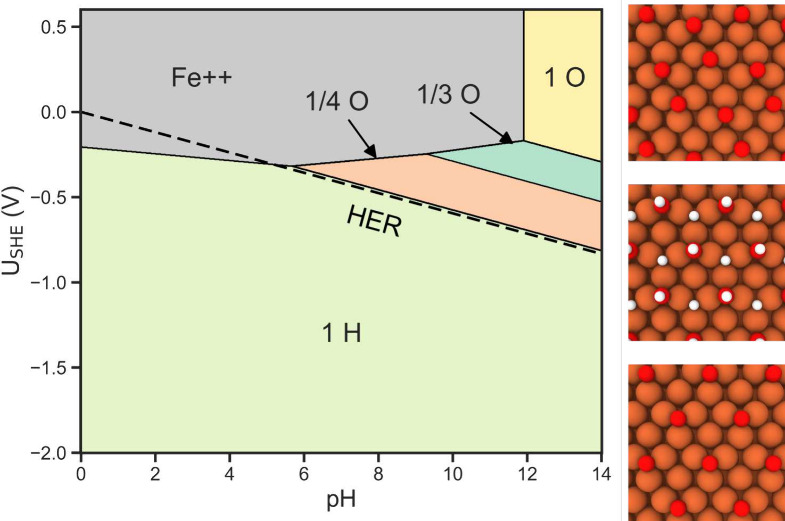

A promising route toward room-temperature ironmaking is electrowinning, where iron ore dissolution is coupled with cation electrodeposition to grow pure iron. However, poor faradaic efficiencies against the hydrogen evolution reaction (HER) is a major bottleneck. To develop a mechanistic picture of this technology, we conduct a first-principles thermodynamic analysis of the Fe110 aqueous electrochemical interface. Constructing a surface Pourbaix diagram, we predict that the iron surface will always drive toward adsorbate coverage. We calculate theoretical overpotentials for terrace and step sites and predict that growth at the step sites are likely to dominate. Investigating the hydrogen surface phases, we model several hydrogen absorption mechanisms, all of which are predicted to be endothermic. Additionally, for HER we identify step sites as being more reactive than on the terrace and with competitive limiting potentials to iron plating. The results presented here further motivate electrolyte design toward HER suppression.

## Introduction

Steel production is responsible for 7–9% of global CO_2_ emissions.^1^ For every ton of steel produced, 2 tons of CO_2_ is released using current methods.^1,2^ In this process, extraction of iron from iron ore using a carbothermic blast furnace contributes 70% of these emissions.^3^ This has led to enormous interest in identifying pathways to decarbonize reduction of iron ore to iron.^4^ Electrochemical approaches offer an energy-efficient route to decarbonization through pairing with renewable electrochemistry. Several industries are adopting this approach, including cement and chemicals production, thereby creating a potential for synergy from concerted electrification across domains.^5−7^

For electrochemical ironmaking, two leading direct electrochemical pathways exist: molten oxide electrolysis (MOE) and electrowinning. MOE is a high-temperature process producing liquid iron and parallels the existing aluminum production process.^8−12^ Despite decades of investment and research, technical challenges remain associated with designing an inert anode that can withstand the harsh thermal and electrochemical conditions needed.^13^ Alternatively, room-temperature electrowinning of iron from iron ore, usually hematite (Fe_2_O_3_), is the second approach where iron plates are grown on the cathode in an electrochemical cell.^14−16^ Rather than using elevated temperatures like in MOE, here Fe–O bond breakage in the iron ore is driven via an applied potential to extract the iron.

Overall, electrowinning can be summarized as a two-step procedure: (i) dissolution of the iron ore and (ii) selective electrodeposition. First, the iron ore is dissolved to extract the iron cations and break the Fe–O bonds. Mechanistically, the exact details of the dissolution process remain an area of active investigation.^14,17−20^ While there has been some suggestion that a bulk conversion is possible for adsorbed iron ore particles, thus bypassing dissolution altogether, this approach is restricted by ore particle size.^21,22^ Thus, we frame the rest of this study within the context of efficient promotion of ore dissolution as the first step. The specific products of the dissolution will be pH dependent (e.g., Fe_2_O_3_ + 6H^+^ ⇌ 2Fe^3+^ + 3H_2_O under acidic conditions^23^). It is worth highlighting, however, that our subsequent discussion and analysis is equally valid for other iron cation sources beyond Fe_2_O_3_ such as Fe_3_O_4_, α-FeOOH, and iron salts (e.g., Fe_2_(SO_4_)_3_).

Following the initial dissolution step, a potential is applied within the cell to drive electrodeposition of the extracted iron cations. This grows high-purity iron plates on the cathode, which can then be further processed to produce other products of interest, such as varying types of steel.^4^ However, this electrodeposition step is hindered by competition with the hydrogen evolution reaction (HER).^14−16^ This challenge bears a resemblance to selectivity challenges observed in many electrocatalysis applications, where details of adsorbate–surface interactions can have an impact, such as the reduction of CO_2_ or N_2_.^24−28^ Thus, efficient and selective electrodeposition is a challenge moving forward.

From a holistic viewpoint, these two steps have fundamentally different environmental requirements. Dissolution is thermodynamically optimized under acidic conditions,^23^ but HER also becomes thermodynamically feasible. On the other hand, alkaline conditions suppress HER but are unfavorable for dissolution. This trade-off leaves an optimization challenge of the electrochemical environment conditions.

Motivated by HER’s suppression under alkaline conditions, it was a major research thrust in the Ultra-Low CO_2_ Steelmaking (ULCOS) project.^14,15,21,22,29^ This was a large collaborative effort from 2004 to 2010 spanning 48 European companies and headed by ArcelorMittal.^29^ Carrying the torch in 2017, the SIDERWIN project, once again coordinated by ArcelorMittal, set out to explore electrowinning of ceramic suspensions in alkaline conditions beyond solely hematite.^16,30−32^ While these projects were monumental in laying the groundwork for this field, the emphasis on alkaline conditions has left broader questions on the nature of the interface under acidic conditions. Recent experimental work has revisited acidic iron electrowinning,^33,34^ as well as the use of pH gradients,^35^ highlighting questions about the underlying electrochemistry.

Thus, general fundamental insights of the electrochemical iron interface is needed to further advance the electrowinning process. While there is extensive surface science literature on the interaction of iron with water and its constituent atoms,^36−45^ as well as established bulk iron–water Pourbaix diagrams,^23,46−48^ a full unified electrochemical mapping in the form of a *surface* Pourbaix diagram has yet to be proposed. In the electrocatalysis field where similar challenges of unification have arisen, surface Pourbaix diagrams have been a powerful tool to predict the surface as a function of electrochemical environment and thereby inform selectivity trends (e.g., chlorine evolution vs oxygen evolution).^49−53^ These diagrams map the thermodynamic preference for surface phase formation as a function of pH and applied potential relative to the standard hydrogen electrode (*U*_SHE_).

In addition to developing a static thermodynamic model of the surface, investigating the dynamics of iron electrodeposition can unlock further insights into the system. Considering similar systems, free energy diagrams have previously enabled predicting the underlying electrochemical growth mechanisms and theoretical overpotentials key to charging Li–air batteries,^54,55^ Zn–air batteries,^56^ and metal anodes.^57^ Moreover, hydrogen embrittlement is a key challenge in ironmaking, and hydrogen absorption has been the subject of several mechanistic studies.^45,58−60^ Continued investigation of hydrogen interactions with iron, but within this electrowinning context in terms of absorption and HER, can shed further light into predicting overall system behavior and plate quality.

In this work, we employ surface Pourbaix diagrams to investigate the thermodynamically most favorable surface phases of O*, OH*, and H* on Fe110 as a function of pH and *U*_SHE_. Interestingly, our results show that there does not exist a region in Pourbaix space where Fe110 is thermodynamically predicted to be both stable toward dissolution and free from any adsorbates. Employing free energy diagrams, we calculate the theoretical overpotentials for iron electrodeposition through terrace (Fe110) and step (Fe210) growth mechanisms. From these diagrams, we emphasize the importance of understanding hydrogen–iron interactions and probe the thermodynamics of absorption and theoretical HER overpotentials. This analysis paves the pathway for potential routes to electrowinning under acidic conditions.

## Methods

To facilitate the thermodynamic analysis presented here, spin-polarized density functional theory (DFT) calculations were conducted using GPAW^61,62^ via the Atomic Simulation Environment package.^63^ Projector-augmented wave potentials were used along with the RPBE exchange-correlation functional.^64^ AutoCat was used to generate all initial structures.^65^ Surface coverage was incorporated via a combination of periodic supercell dimensions and explicit inclusion of the adsorbates. All terrace slabs were four layers (unless otherwise noted) and stepped slabs eight layers. Additional computational details are provided in the Supporting Information.

Modeling this aqueous environment, the following water oxidation reactions can occur on the surface forming either O* or OH*:
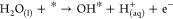
1
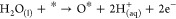
2where * indicates an unoccupied surface site and X* is an X molecule adsorbed on the surface.

Additionally, the formation of H* species on the surface can occur through a Volmer process, the first step of HER:
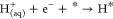
3

In predicting the most thermodynamically favorable surface phases of Fe110, the energetics of the above processes are key. Calculation of these free energies are done by invoking the computational hydrogen electrode (CHE).^66^ This approach sets the reference potential to be that for which the reaction  is in equilibrium. From this choice, surface phase formation energies as a function of pH and *U*_SHE_ can be extracted, taking into account the number of consumed or produced electrons and protons. For example, the free energy change upon formation of an O* phase formed via eq 2 can be expressed as
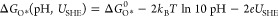
4where *k*_B_ is the Boltzmann constant, *e* is the fundamental charge, and *ΔG*_O*_^0^ is the energy upon formation at 0 V (vs SHE) and pH = 0. *ΔG*_O*_^0^ is obtained directly from DFT, and similar energy expressions can be derived for surface phases involving H* and OH*. Moreover, to allow for comparisons across monolayer (ML) coverages, these calculated energies are subsequently normalized to be per surface atom. We refer the reader to the Supporting Information for more details on the calculation of these energetics.

These energies are not only governed by the strength of adsorbate interactions with Fe110 relative to their reference phases but also adsorbate coverage and patterns on the surface. At higher coverages, steric effects can become more pronounced, and at a given coverage, there may exist multiple configurations. As will be discussed in greater detail in the following section, we thus modeled several supercell sizes, number of adsorbates, and adsorbate placements to obtain a variety of surface phases.

In addition to the surface phases, stability of the Fe110 surface is also important and we consider the following iron dissolution reaction:
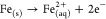
5

Following a similar logic as for the adsorbate phases, the energy change upon dissolution at *a*_Fe^2+^_ = 1 can be expressed as
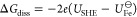
6where *U*_Fe_^⊖^ is the redox potential for this process at standard conditions (−0.45 V vs SHE^67^). It is worth noting that as this process does not produce or consume H^+^, it is independent of the pH of the environment. Dissolution of the iron surface forming HFeO_2_^–^ is also possible, but we assume this will have a considerable kinetic barrier and thus exclude it from our analysis.

Free energy diagrams for iron deposition were calculated by sequentially adding iron atoms either on the terrace of a Fe110 slab or a Fe210 stepped surface. Energy differences from depositing a single Fe atom are calculated via the following equation:
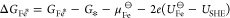
7where *G*_Fe*_ is the total energy after deposition, G_*_ is the total energy before deposition, and μ_Fe_^⊖^ is the energy per bulk iron atom. For these iron deposition calculations, the vibrational contributions to energy were neglected. To ensure consistent k-point sampling between the surface and bulk iron energy cells, the linear fitting method^68^ was used to calculate μ_Fe_^⊖^. For fitting this value, we used primitive surface slabs with odd numbers of layers ranging from 3 to 19 to converge the bulk energy to within 0.05 eV. We refer the reader to the Supporting Information for additional methodological details including computational parameters and calculating energies with CHE.

## Results and Discussion

### Surface Phases of Fe110

To model the iron surface at the cathode in an electrowinning cell, we use a body-centered cubic (bcc) Fe110 slab model. Previously, this surface facet was shown to possess the lowest surface energy.^69−71^ In addition, the 110 facet has been previously identified for electrodeposited iron films using both atomic force microscopy (AFM) and X-ray diffraction (XRD).^72−74^ Moreover, even with electrodeposition on a face-centered cubic substrate, a bcc transition was observed upon increasing layers deposited, and the 110 facet was observed to emerge.^73,74^ Thus, through the selection of this bcc structure, the analysis presented in this work focuses on growth after the initial nucleation stage. As the environment is aqueous, we consider the energetics of water oxidation and Volmer processes to form OH*, O*, and H* on the surface. These adsorbates form surface phases based on their coverage and pattern on the Fe110 terrace. The energies of forming these phases are not only governed by the strength of adsorbate–surface interactions but also adsorbate coverage and patterns on the surface. By modeling several supercell sizes, number of adsorbates, and adsorbate placements we obtain a variety of surface phases that can be considered.

Identifying the most favorable adsorption sites for O*, H*, and OH*, we calculate adsorption energies at 1/4 ML coverage by relaxing the adsorbates at all unique surface sites on the 110 terrace facet (Figure S1): hollow, long bridge, short bridge, and ontop. These calculated adsorption energy values are given in Table 1. It is observed that for H* at this coverage, the hollow site yielded the most negative adsorption energy. Much of the previous literature, both computational and experimental, identifies either the hollow or long bridge as the most favorable site,^36−44^ and we highlight the small geometric distance distinguishing these two sites. Similarly, OH* and O* were also observed to prefer the hollow site, and for all other coverages on the terrace, we assume that this site will be most favorable.

**Table 1:** Adsorption Energies of H, O, and OH at 1/4 ML Coverage on Fe110

	*ΔG*^0^ (eV)			
adsorbate	hollow	on top	long bridge	short bridge
H	–0.60	0.40	–0.29	–0.42
O	–0.99	0.82	–0.67	–0.52
OH	–0.59	0.31	–0.30	–0.39

To develop as complete a picture of the surface as possible, we modeled several different surface coverages and phases. In the following paragraphs we discuss the phases considered, with particular emphasis on coverages that have multiple different patterns.

**Figure 1:**
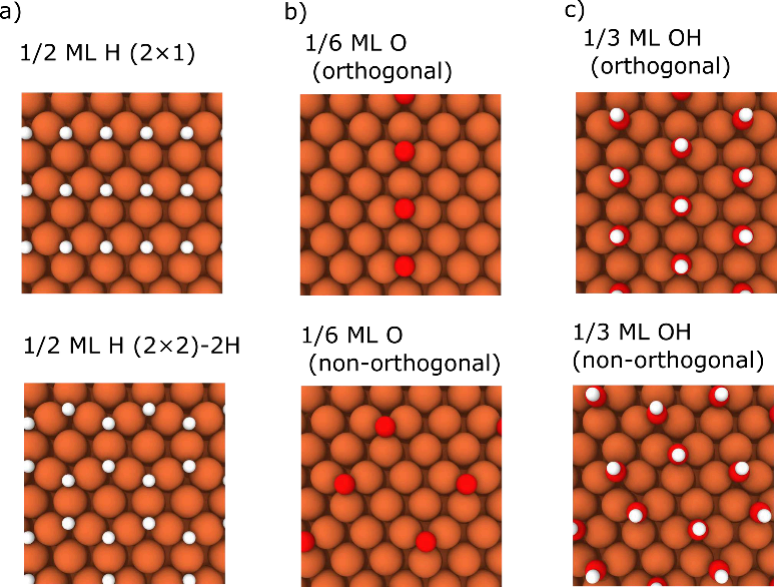
(a) Two surface configurations of H at 1/2 ML coverage (top: 2 × 1; bottom (2 × 2)-2H). (b) Two surface configurations of O at 1/6 ML coverage (top: orthogonal unit cell; bottom: nonorthogonal unit cell). (c) Two surface configurations of OH at 1/3 ML coverage (top: orthogonal unit cell; bottom: nonorthogonal unit cell).

At 1/2 ML coverage for all three adsorbates, we considered two phases, as described by Jiang and Carter:^37^ (2 × 1) and (2 × 2)-2H. The former has a rectangular pattern on the surface whereas the latter forms a hexagonal pattern, as shown in Figure 1a. H* and O* show a slight preference toward the hexagonal configuration at 1/2 ML coverage, in agreement with previous work.^37,75^ OH* also shows a slight preference toward the hexagonal phase at this coverage. On a per-surface atom basis, this preference is at most a difference of approximately 30 meV for the three adsorbates.

At 1/6 ML and 1/3 ML coverages, we considered two different configurations for each. One is formed by using an orthogonal cell where the other uses a conventional nonorthogonal cell in the *xy* plane (Figure 1b,c). H* shows a preference toward the orthogonal configuration at both 1/6 ML and 1/3 ML coverage but by only ∼5 meV/surface atom. The differing behavior of H* may be rationalized by decreased steric effects, as evidenced by the vertical OH* orientations on the surface. Both OH* and O* prefer the pattern formed via the nonorthogonal cell at these coverages.

In addition to these single adsorbate phases, we also considered mixed OH* + H* phases, as they can be formed via the chemical dissociation of water. At a total of 1/2 ML, we model both configurations ((2 × 1) and (2 × 2)-2H) in the mixed phase at this coverage with 1/4 ML OH* + 1/4 ML H* (Figure S3). Again, we observe minimal energetic difference, with a marginal preference toward the hexagonal phase of ∼5 meV/surface atom. We also model the higher coverage mixed phase 1/2 ML OH* + 1/2 ML H* to fully cover the surface (Figure S3). Interestingly this phase is predicted to be thermodynamically stable, unlike the 1 ML O* and 1 ML OH* phases, possibly through the OH–H hydrogen bonding as evidenced from the induced tilt of the OH moieties. Visualizations of all surface phases considered in this work are aggregated in Figures S2 and S3.

### Mapping the Surface Pourbaix Diagram

**Figure 2:**
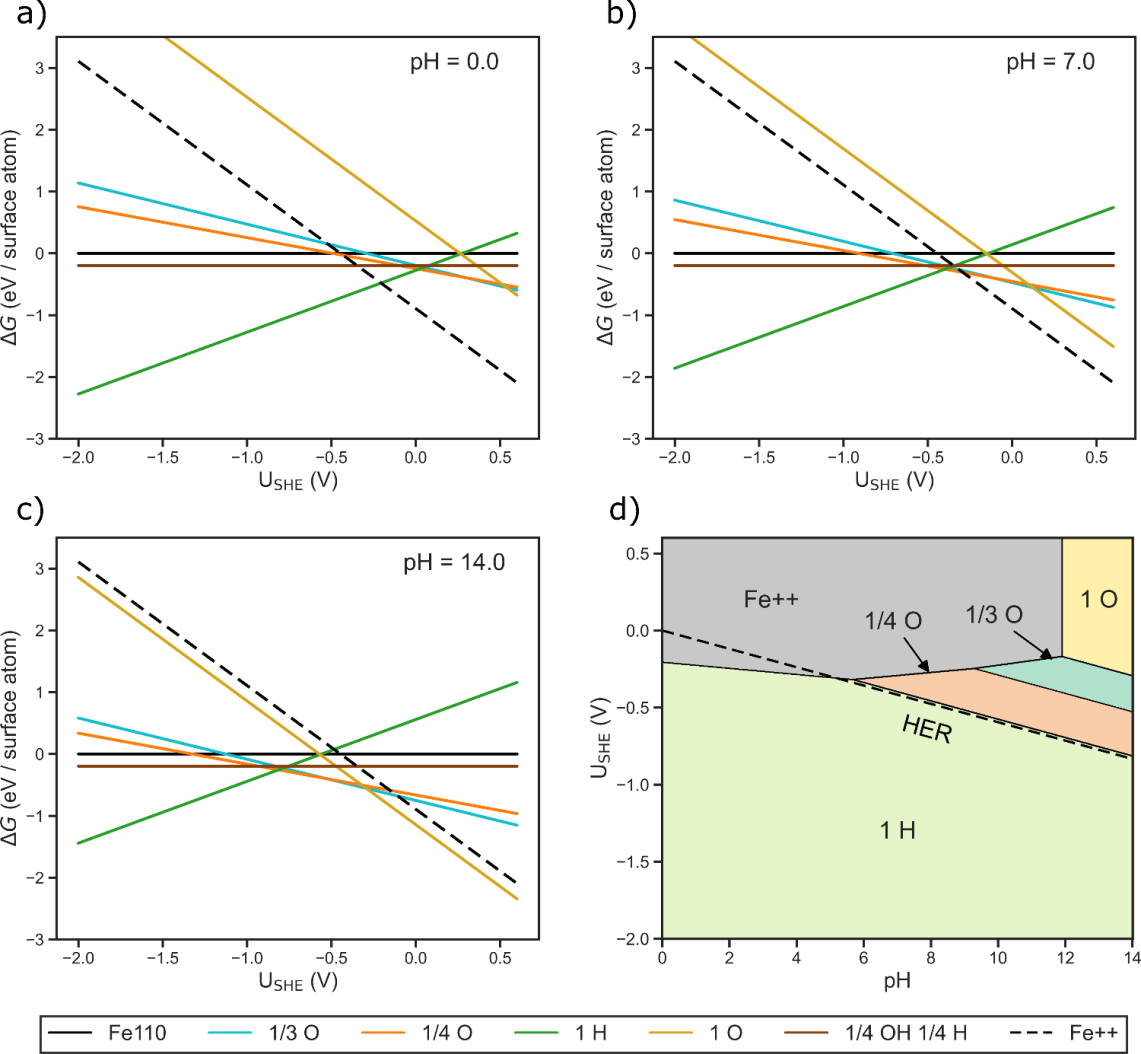
Surface Pourbaix slices for pH values of (a) 0.0, (b) 7.0, and (c) 14.0. (d) Full surface Pourbaix diagram.

Aggregating the above calculations and corresponding pH and *U*_SHE_ dependencies, we visualize *ΔG* as a function of *U*_SHE_ for a selection of phases in Figure 2. In the acidic and neutral regimes (Figure 2a,b), we observe that for negative applied potentials the surface is predicted to be driven toward full hydrogenation. Increasing the potential is predicted to lead to dissolution of the Fe110 surface beyond approximately −0.20 V (vs SHE) at pH = 0.0 and −0.28 V (vs SHE) at pH = 7.0.

Moving into the alkaline regime with a pH of 14.0 (Figure 2c), it is predicted that oxygenated surface phases will emerge that prevent dissolution. Previous targeted surface science works have observed the formation of oxygen covered phases on Fe110, thereby demonstrating a general predisposition for the formation of these phases.^38,39^

Continuing this analysis and treating pH as a continuous variable, we plot the surface Pourbaix diagram in Figure 2d. This diagram maps the surface to predict the thermodynamically most favorable phases as a function of electrochemical environment. Within the context of iron electrodeposition, this enables prediction of surface coverage of the grown iron plates under relevant electrochemical conditions. As this approach does not incorporate changes in the bulk, the pH and *U*_SHE_ range is selected to mostly coincide with stable Fe from the bulk Pourbaix diagram.^23,46−48^ Throughout this space there is no region where a clean Fe110 surface is thermodynamically preferred. For most negative potentials, 1 ML H* is the favored phase. At potentials that are near 0 V (vs SHE) or are positive, either O* phases are predicted to form or dissolution of the surface is favored depending on the pH. We highlight that much of these O phases overlaps with oxide phases in the bulk Pourbaix, which implies bulk changes could occur in the iron. With the aim of promoting pure iron growth, and identifying promising environmental conditions for this process, optimizing for low coverage surface phases may be beneficial for ease of deposition due to increased exposed surface area. Thus, this diagram aligns with previous motivation toward exploring alkaline conditions.

Focusing on the acidic to neutral region, we additionally calculate a surface Pourbaix diagram for the Fe210 step modeling 1/5 ML, 3/5 ML, and 1 ML coverage of the steps by O and H. Since the terrace surface Pourbaix diagram predicts a drive toward 1 ML H coverage, we cover the terrace region of this step model with hydrogens (Figure S6). Here we predict that for all regions where the surface is predicted to be stable, there will be a thermodynamic drive toward keeping the step free from adsorbates (Figure S7).

We note that this analysis does not include solvation in calculating the thermodynamics of these surface phases. Through hydrogen-bonding, the water molecules from the aqueous environment can stabilize the surface adsorbates. For example, the water layer structure on metallic interfaces is an area of extensive study.^76−79^ Of particular relevance here, OH has been observed to incorporate itself into the water layer network on Pt which can stabilize it on the surface.^80^ On the other hand, solvation has a negligible effect on atomic O adsorbates.^66^

To gauge how this effect may change the physical conclusions of our model, we focus on the neutral to acidic region and applied potentials leading to a stable surface. Here we investigate the stabilization required to displace the 1 ML H phase as most favored. Without solvation, the most stable phase of pure OH is the 1/4 ML OH phase, where the most stable mixed phase involving OH is the 1/4 ML OH + 1/4 ML H phase. Here we calculate the difference between the formation energy of these phases and the 1 ML H phase as a function of pH and applied potential. At pH = 0, the OH molecules would need to be stabilized by 10.51 eV for the entire 1 ML H region to be replaced by 1/4 OH. The difference at this pH is slightly smaller for the mixed 1/4 ML OH + 1/4 ML H phase, 8.32 eV, but remains substantial. For the OH phases to replace part of the 1 ML H phase, i.e., up until −0.5 V, the differences are 4.32 and 2.32 eV for the pure and mixed OH phases, respectively. Moving into the neutral regime, the difference is further decreased but there remains a large gap to overcome. At pH = 7 and for the OH phases to become more thermodynamically favorable until −0.5 V, the mixed phase is 0.65 eV less stable than 1 ML H. For context, OH is stabilized by 0.33 eV on Pt by the water layer,^66^ implying that solvation alone is unlikely to significantly alter our predicted surface Pourbaix diagram for the acidic to neutral regimes.

It is also worth noting that this analysis does not incorporate kinetics and is strictly based on thermodynamics. Thus, large activation barriers may hinder the formation of the predicted thermodynamically favored phases under given conditions. In light of this, this diagram represents phases the surface will be driven toward. Moreover, our analysis is restricted to including the surface phases calculated in the previous section. In reality, intermediate coverages or combinations of these surface phases may coexist on the surface. Methods toward addressing this limitation is an active area of research^53,81^ and is beyond the scope of this work.

### Theoretical Overpotentials for Iron Electrodeposition

To understand the most relevant region of the surface Pourbaix for iron electrodeposition (the reverse reaction of eq 5), we next calculate the theoretical limiting potentials and overpotentials associated with plating. The theoretical limiting potential *U*_L_ is the least negative applied potential for which all steps are downhill in a free energy landscape for a given reaction mechanism. Taking the difference between this limiting potential and the equilibrium potential *U*_Fe_^⊖^ yields the theoretical overpotential.

For iron electrowinning, the reaction of interest is electrodeposition. The energetics of this process govern the efficiency of the growth of pure iron plates. In this work we consider two main growth mechanisms: (i) island nucleation on a Fe110 terrace and (ii) kink formation at a Fe210 step. Modeling both of these mechanisms was performed by sequentially placing one iron atom at a time until the terrace or step is reproduced. As a simplification, we model both of these surface models without the presence of H*, O*, and OH* adsorbates. Thus, the calculated potentials in this section can be interpreted as a scenario where adsorbate formation is blocked either kinetically or through electrolyte design. Adsorbates present on the surface are expected to generally have a detrimental impact on these overpotential values and possibly plate composition.

**Figure 3:**
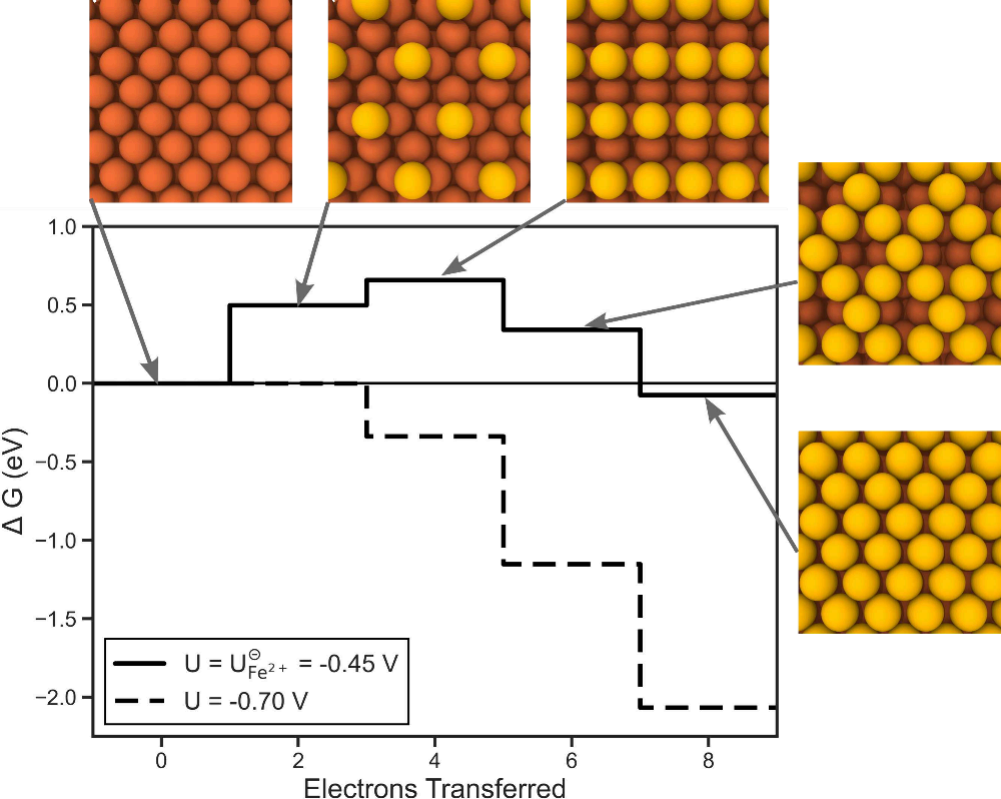
Free energy diagram for sequential electrodeposition of iron atoms on an Fe110 terrace. Yellow atoms in the atomic images highlight the iron atoms deposited so far. The supercells have been repeated to visualize the overarching patterns.

We first model the growth mechanism stemming from island nucleation on a terrace (Figure 3). In this case, iron atoms are placed until another full layer of iron is deposited on the surface. At the standard reduction potential for iron, the first two iron deposition steps are endothermic and the latter two steps are exothermic. Ideally, placement of the final iron should return the energy to 0 eV as the terrace has been reproduced. However, the observed deviance of 0.07 eV is close to our applied convergence criteria for the bulk energy, 0.05 eV. Moreover, this deviation may be a consequence of differing coordinations of the reference bulk and surface iron atoms, as observed in other metals.^57^ Applying limiting potential analysis to this free energy diagram, the most uphill step in this growth mechanism is placement of the first iron atom with a value of 0.50 eV. The limiting potential is calculated by  referenced to SHE. Since the theoretical overpotential is the difference between the standard reduction potential and limiting potential, it is calculated by . Therefore, this initial island nucleation step is the potential determining step, yielding a limiting potential *U*_L_ of −0.70 V vs SHE. Propagating this quantity to obtain the theoretical overpotential, we calculate this value to be 0.25 V for deposition on a terrace.

Further investigating this terrace growth mechanism, we point out that for placing the second iron atom on the surface there are two symmetrically unique configurations (Figure S5). Their energies are comparable with values of 0.66 and 0.71 eV. Since this second step is not potential determining, both deposition paths are thermodynamically accessible when the potential applied is at least *U*_L_.

**Figure 4:**
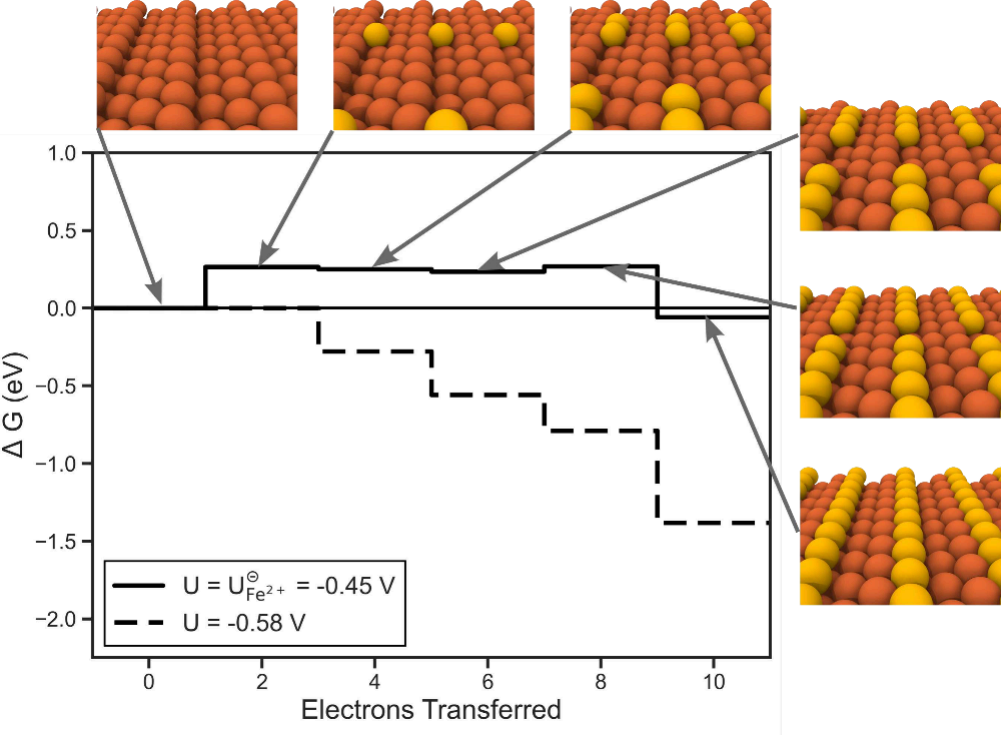
Free energy diagram for sequential electrodeposition of iron atoms at an Fe210 step. Yellow atoms in the atomic images highlight the iron atoms deposited so far. The supercells have been repeated to visualize the overarching patterns.

Turning now to electrodeposition at steps, we next investigate growth via kink formation. Here we use the Fe210 surface facet as our model. In alignment with previous low-energy electron diffraction experiments, we observe significant relaxation from the bulk with comparable interlayer spacings as shown in Table S1.^82^ Applying this model for the step growth mechanism at the standard reduction potential (Figure 4), the first step is energetically uphill to nucleate the kink with subsequent steps relatively flat in energy. Thus, as in growth at the terrace, we observe the first deposition step to be the PDS. In contrast to growth on the terrace, however, we observe the magnitude of this PDS to be relatively smaller (0.26 eV) leading to a *U*_L_ of −0.58 V vs SHE. The theoretical overpotential for growth at the step site is calculated to be 0.13 V.

Comparing the theoretical overpotentials between growth at the terrace and step sites, we predict through this idealized model for growth to mainly occur via kink formation. This is in alignment with previous conclusions drawn for electroplating of other metals.^57^ However, we reiterate that this neglects solvation effects through adsorbate formation as well as diffusion kinetics which could influence these growth mechanisms.

It is worth highlighting the limitations of this growth model. With the restricted cell size of our model, it currently focuses on the overall growth energetics. Previous epitaxial growth investigations have observed island formation followed by coalescence with increasing coverage.^83−85^ Specifically for electrodeposition, layer-by-layer growth with relatively flat deposits after island formation was also reported using this method.^73,86^ Thus, if island clusters are to form as an intermediate growth stage, our model will not capture this detail. Further investigations into expanding upon this model will be the subject of future investigation.

Additionally, we note that the calculated limiting potentials of both terrace and step site mechanisms both lie within the 1 ML H region of the surface Pourbaix diagram (Figure 2d). This observation implies that potentials required to electroplate the iron cations from solution will also thermodynamically drive hydrogen adsorbate formation. In other words, iron deposition may proceed on Fe110 in the presence of hydrogen adsorbates thereby introducing the possibility for more complicated interactions and growth mechanisms. A coupled analysis further investigating the interplay of hydrogen as the layer grows is necessary to gauge the likelihood of more intertwined deposition mechanisms. Such a comprehensive analysis is beyond the scope of this initial work but is another avenue for future study.

### Hydrogen Evolution and Absorption

As the surface phases associated with hydrogen formation are most thermodynamically favorable under the required conditions for iron electrodeposition, we further investigate these iron–hydrogen interactions. Experimental investigations into the interplay of iron and hydrogen have proposed as early as the 1960s that the hydrogen adsorption state can lead to two further interaction modes: hydrogen absorption into the bulk and HER.^87^ Investigating each of these processes, we consider both terrace and steps to gauge impact stemming from different coordinations of the surface iron atoms. Previous work has highlighted increased reactivity of iron steps with both nitrogen and water,^44,88^ emphasizing the interplay between surface morphology and surface–adsorbate interactions.

**Figure 5:**
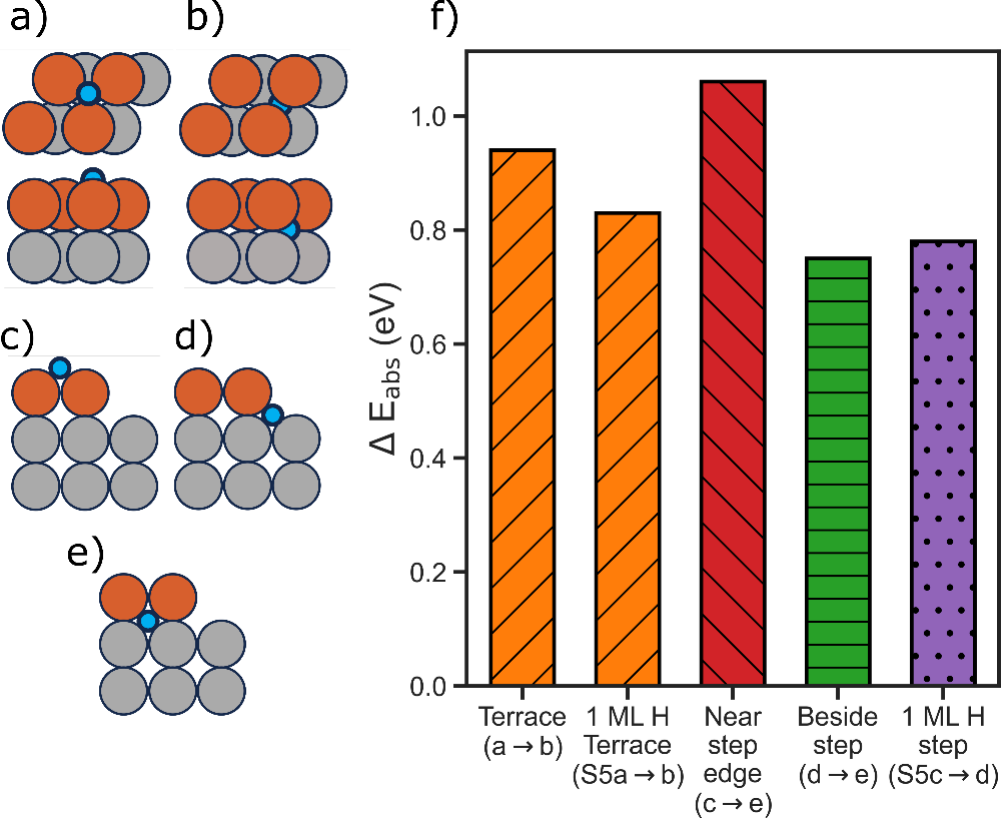
Schematics illustrating absorption structures with hydrogen placed (a) at terrace surface hollow site (top and side views), (b) below terrace short bridge site (top and side views), (c) hollow site near step edge, (d) hollow site adjacent to step, and (e) below short bridge site near step edge. Outermost surface iron atoms are brown, subsurface iron atoms are gray, and hydrogen atoms are blue. (f) Summary of calculated absorption enthalpies for all paths considered, color-coded by mechanism.

For absorption, the hydrogen will first adsorb onto the surface at the hollow site via a Volmer process (Figure 5a). On the terrace, to allow for greater reconstruction of the slab to accommodate hydrogen entry into the subsurface, we increase the number of layers of our model from four to five with the top three layers free to move. In the *x*–*y* dimensions the terrace supercell model is kept at 2 × 2. With this thicker slab, we again relaxed hydrogen on the surface at the hollow site and calculate an adsorption enthalpy of −0.91 eV. To identify the most stable hydrogen site upon entry into the slab, we relaxed the hydrogen in the subsurface underneath the top site, hollow site, long bridge, and short bridge. For initial placement in the subsurface below the hollow and long bridge sites, the hydrogen atoms were spontaneously ejected to the surface. Calculating the enthalpies referenced to gas phase for placement under the short bridge and top site, we obtain values of 0.03 and 0.11 eV, respectively. This thermodynamic preference toward hydrogen absorption beneath the short bridge is in qualitative agreement with earlier reports,^60^ and the configuration is illustrated in Figure 5b. From these values, we calculate the enthalpy for terrace absorption to be 0.94 eV. This finding is in good agreement with previous reports of 0.98 eV^60^ and 1.00 eV.^59^ Moreover, we calculate the enthalpy of absorption to below the short bridge for 1 hydrogen from the fully hydrogenated Fe110 terrace (Figure S5a,b). We calculate this process to be slightly less endothermic (0.83 eV) than for the clean surface, indicating that neighboring hydrogens may help encourage this absorption process.

Extending this analysis to include entry at step sites, we evaluated two scenarios for the clean 210 step: (i) absorption from near the step edge (Figure 5c) and (ii) absorption from adjacent to the step (Figure 5d). In both mechanisms explored, the hydrogen diffuses to below the short bridge near the step edge since it was most favorable in the terrace (Figure 5e). Starting from a hollow site near the step edge, we calculate an enthalpy of absorption to be 1.06 eV. Alternatively, if absorption starts from a hollow site directly adjacent to the step, we calculate an enthalpy of absorption of 0.75 eV. While this latter case exhibits a less uphill enthalpy for absorption, calculation of the kinetic barriers is required to determine whether it is predicted to be the more likely absorption mode. We additionally consider where the step is hydrogenated on the terrace and hydrogen enters from the step (Figure S5c,d). This process is less endothermic than the mechanism near the step edge but is comparable to entry from beside the step (0.78 eV). Similarly to the step edge, this indicates that nearby hydrogens may help hydrogen entry in some configurations.

As all absorption mechanisms investigated are endothermic (Figure 5f), there is an initial barrier for hydrogen entry into the pristine iron surface. However, defects and vacancies have been identified as key toward hydrogen embrittlement through the introduction of traps,^45^ and after initial absorption into the bulk, the hydrogen atoms are anticipated to exhibit increased mobility.^59,60^

In addition to entering the iron, adsorbed hydrogen on the surface may alternatively further react to form H_2_ via HER. After the initial Volmer step, HER may proceed via a Heyrovsky process as follows:
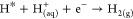
8

**Figure 6:**
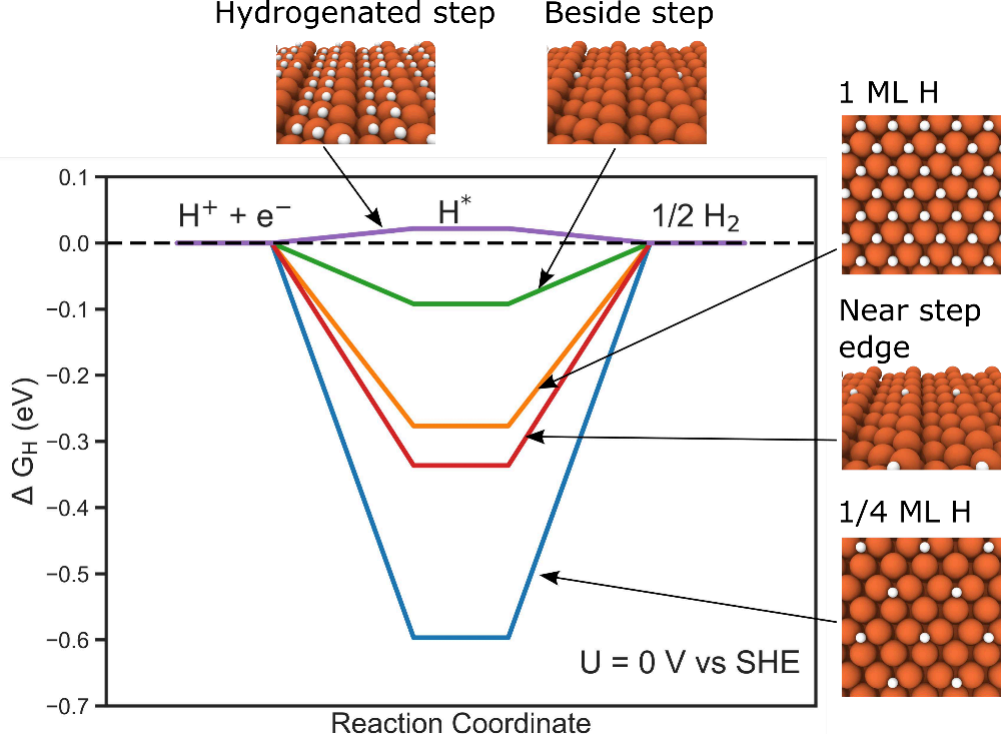
Free energy diagrams for HER on the terrace sites (1/4 and 1 ML coverage), beside the step, near the step edge, and on a hydrogenated step. Values closer to zero are predicted to have higher HER activity. All sites are predicted to compete with iron electrodeposition

For HER, *ΔG*_H_ ∼ 0 has generally been used as a descriptor toward catalytic activity.^89,90^ We thus calculate the adsorption energy of hydrogen on the terrace and several step configurations, which are summarized in Figure 6. All subsequent values are calculated for pH = 0. Starting with the terrace, our calculated adsorption energies used in constructing the surface Pourbaix are −0.60 eV/H atom and −0.28 eV/H atom for 1/4 and 1 ML, respectively. Of these two phases, we predict full coverage to be generally more dominant by the surface Pourbaix (Figure 2d). In comparison to the calculated limiting potential for iron growth on the terrace, −0.70 V, the −0.42 V limiting potential for HER is predicted to be competing with iron plating, as expected.

We next investigate HER at the Fe210 step by considering multiple possible active sites in the vicinity. Initially placing the hydrogen between two step iron atoms, the system relaxed to one of two sites as described in the absorption discussion: (i) beside the step above a hollow site (Figure 5c) or (ii) near the step edge (Figure 5d). For each of these configurations we calculate the ZPE to obtain more accurate vibrational corrections (Table S3). In addition to these two sites, we also relaxed the system starting with hydrogen singly coordinated to a step iron, and the system converged to a configuration beside the step but over a top site. Performing vibrational analysis on this configuration, however, revealed an imaginary mode from oscillating parallel to the step edge indicating it is metastable. Thus, we focus the analysis on the two stable sites.

The corresponding adsorption energies were calculated to be −0.09 eV/H atom beside the step and −0.34 eV/H atom near the step edge. With these values closer to zero than on the terrace, this enforces the viewpoint that the step sites may be more reactive than the terrace. Comparing these adsorption energies with the step growth limiting potential, −0.58 V, competition between HER and iron deposition is again predicted.

Under neutral to acidic conditions, the surface will be driven toward forming a hydrogen overlayer on the terrace. Thus, we additionally calculated the HER landscape on the 210 step where the terrace portion is covered by hydrogen (Figure 6). In this scenario, the hydrogen is stabilized on the step edge at the bridge between two step iron atoms. With the introduction of the nearby hydrogen overlayer in our model, the hydrogen binding at the step is weakened further to 0.02 eV/H atom. Therefore, hydrogen coverage on the terrace sites increases the reactivity of the steps toward HER even further.

As an alternative to the Heyrovsky step, two adsorbed hydrogens may chemically recombine via the Tafel process, 2H* → H_2(g)_. Previous experimental studies into the mechanism have observed this Tafel step to be more prevalent at lower overpotentials and Heyrovsky at moderate to higher overpotentials.^87,91^ Thus, our thermodynamic analysis using the Heyrovsky step may be interpreted as an upper bound on HER performance. As the Tafel step is a largely kinetic process, a kinetic analysis is required for a more comprehensive view of HER on iron. Extending our analysis here to incorporate kinetics alongside hydrogen coverage will be the subject of future study. However, the results here emphasize HER as a challenge toward the efficient electrowinning of iron and requires electrolytes that can suppress it (e.g., water in salt electrolytes^92,93^).

## Conclusions

In summary, we modeled several surface phases on Fe110 ranging in coverage from 1/6 to 1 ML and including mixed adsorbate phases. Using the associated calculated energies of these phases, we generated surface Pourbaix diagrams and observed that there is a thermodynamic preference for adsorbates in all regions of surface stability. Calculating the theoretical overpotentials for iron deposition at the terrace and step sites, we predict a thermodynamic preference toward growth at the steps. Further investigating the hydrogen–iron interactions we calculate enthalpies for multiple absorption processes via the terrace and step, all of which determined to be endothermic. In addition, we calculate terrace and step HER limiting potentials and reinforce the challenge selectivity. The analysis here presents an important step toward mapping the aqueous electrochemical interface of iron and could shape the design of sustainable ironmaking through acidic electrowinning.

## Data Availability

All structures and data are available at the following GitHub repository: https://github.com/BattModels/iron_electrowinning_mapping.

## Supporting Information Available

Additional discussion on computational details, calculating energetics with the computational hydrogen electrode, visualizations of all considered surface phases, and schematics for hydrogen absorption mechanisms for hydrogenated surfaces (PDF)
