# Supplementary material for: Electrowinning for Room-Temperature Ironmaking: Mapping the Electrochemical Aqueous Iron Interface
Source: J Phys Chem C Nanomater Interfaces. 2024 Aug 22;128(35):14611–20. doi: 10.1021/acs.jpcc.4c01867 (PMC11382279; doi:10.1021/acs.jpcc.4c01867)
Supplement: Supplementary file 1 — jp4c01867_si_001.pdf [file jp4c01867_si_001.pdf]

# Supporting Information for Electrowinning for Room-Temperature Ironmaking: Mapping the Electrochemical Aqueous Iron Interface

Lance Kavalsky[†,‡] and Venkatasubramanian Viswanathan[*,†,‡]

†*Department of Mechanical Engineering, Carnegie Mellon University, Pittsburgh, Pennsylvania 15213, USA*

‡*Department of Mechanical Engineering, University of Michigan, Ann Arbor, Michigan 48109, USA*

E-mail: venkvis@umich.edu

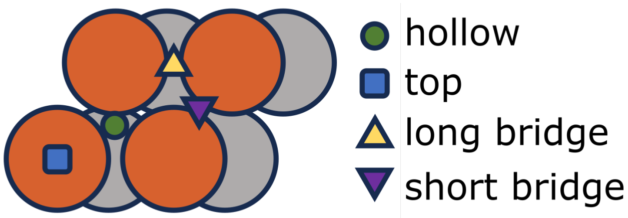


Figure S1: All symmetrically unique sites on a bcc110 surface. Brown atoms are at the surface, and grey are in the subsurface

**Table S1: Comparison of interlayer spacings of Fe210 step model to experimental values obtained via LEED [179].** $d_{ij}$ **corresponds to the distance in the z-axis between layers** $i$ **and** $j$ **and** $a_{ij}$ **is the distances in the x-axis (see Fig. 5 from Ref. [179] for more details)**

| | This work (Å) | Experiment (Å) [179] |
|---|---|---|
| $d_{12}$ | 0.522 | 0.50 ± 0.03 |
| $d_{23}$ | 0.612 | 0.57 ± 0.03 |
| $d_{34}$ | 0.765 | 0.75 ± 0.03 |
| $d_{45}$ | 0.593 | 0.61 ± 0.03 |
| $a_{12}$ | 1.991 | 2.06 ± 0.05 |
| $a_{23}$ | 1.972 | 1.95 ± 0.05 |
| $a_{34}$ | 1.917 | 1.92 ± 0.05 |
| $a_{45}$ | 1.958 | 2.00 ± 0.05 |

# Additional Computational Details

For all DFT calculations a target grid spacing of 0.16 Å was applied with Fermi-Dirac smearing of 0.05 eV to help improve self-consistent field convergence. As adsorbates were placed on a single side of the slab, a dipole correction was applied in the z-direction to correct for the asymmetry. Every terrace slab consisted of four layers, with the bottom two layers fixed to their bulk positions (unless otherwise noted). To model a step, an Fe210 1×5 supercell slab was used consisting of 8 layers with the bottom 2 rows held fixed to their bulk positions. All structures were relaxed until the maximum force was less than 0.05 eV/Å.

To model an assortment of adsorbate coverages and patterns, periodicity in the xy-plane, varying dimensions of supercells, and number of explicit adsorbates were leveraged. Accordingly, k-meshes were chosen for each slab size (Table S2). We note that for the 3×2

slab, we considered both orthogonal and non-orthogonal slabs.

**Table S2: k-mesh used for each supercell considered in this study**

| supercell dimensions | k-mesh |
|---|---|
| 1×1 | 12×12×1 |
| 2×2 | 6×6×1 |
| 3×2 (orthogonal) | 4×8×1 |
| 3×2 (non-orthogonal) | 4×6×1 |
| 1×5 (210 stepped slab) | 5×2×1 |

## Calculating Energetics with the Computational Hydrogen Electrode

To calculate the Gibbs Free energies for each phase we set the reference electrode to be the computational hydrogen electrode (CHE).[1] This is defined as the potential for which the reaction $H^+ + e^- \rightleftharpoons \frac{1}{2}H_2$ is in equilibrium. This choice is convenient as it avoids the need to calculate $\mu_{H^+(aq)}$ since it sets $\mu^0_{H^+(aq)} = \frac{1}{2}\mu^0_{H_2(g)}$ at pH = 0. Furthermore, the chemical potential for the electron can then be expressed as $\mu_{e^-} = -eU_{SHE}$ where $U_{SHE}$ is the applied potential relative to the standard hydrogen electrode (SHE).

Additionally, in line with the original CHE paper,[1] we assume that the water is at its liquid-vapor equilibrium (p = 0.035 bar and T = 298 K) and use the entropy at this state which sets $\mu_{H_2O\,(l)} = \mu_{H_2O\,(g)}$. In this work we also neglect the electric field.

With the above choices and approximations, we now have the tooling to calculate adsorption free energies for the formation of surface phases involving H*, O*, and OH*. The energy changes for the reactions described in Eq. 1-3 of the main text at 0 V vs SHE and pH = 0 can be expressed as below:

$$\Delta G^0_{\mathrm{O}^*} = G_{\mathrm{O}^*} + \mu_{\mathrm{H_2(g)}} - G_* - \mu_{\mathrm{H_2O\,(g)}} \tag{S1}$$

$$\Delta G^0_{\mathrm{OH}^*} = G_{\mathrm{OH}^*} + \frac{1}{2}\mu_{\mathrm{H_2(g)}} - G_* - \mu_{\mathrm{H_2O\,(g)}} \tag{S2}$$

$$\Delta G^0_{\mathrm{H}^*} = G_{\mathrm{H}^*} - G_* - \frac{1}{2}\mu_{\mathrm{H_2(g)}} \tag{S3}$$

where $G_{X^*}$ is the energy of the modeled slab with 1 $X$ adsorbed and $G_*$ is the energy of the clean slab. For systems where multiple adsorbates are explicitly modeled on the surface, the coefficients for the reference states (e.g. $\mu_{\mathrm{H_2(g)}}$) are adjusted accordingly. Furthermore, this same logic is used for considering mixed phases. For example, the energy for forming a mixed OH* and H* phase can be expressed by combining the reactions described in Eq. 1,3:

$$\Delta G^0_{\mathrm{OH}^*+\mathrm{H}^*} = G_{\mathrm{OH}^*+\mathrm{H}^*} - G_* - \mu_{\mathrm{H_2O\,(g)}} \tag{S4}$$

These Gibbs free energies are calculated by applying vibrational corrections to the DFT total energies as follows:

$$G = E_{\mathrm{DFT}} + \mathrm{ZPE} - \mathrm{TS} \tag{S5}$$

where $E_{\mathrm{DFT}}$ is the DFT total energy, ZPE is the zero-point energy and TS is the entropic term. For adsorbates we calculate the ZPE in the harmonic approximation at 1/4 ML coverages and assume this will be approximately independent of coverage. We also assume configurational entropy is negligible on the surface. On the other hand for the gas phase references, the ZPEs were calculated in the ideal gas limit and entropies taken from previously established experimental values at the liquid-vapor equilibrium.[1] The corrections are summarized in Table S3.

Using the chemical potential of the electron and a pH correction, we can use these

**Table S3: Vibrational corrections to adsorbates in eV**

| | TS | T$\Delta$S | ZPE | $\Delta$ZPE | $\Delta$ZPE - T$\Delta$S |
|---|---|---|---|---|---|
| $H_2O$ | 0.67 | 0 | 0.57 | 0 | 0 |
| $H_2O \rightarrow OH^* + \frac{1}{2}H_2$ | 0.21 | -0.47 | 0.46 | -0.11 | 0.36 |
| $H_2O \rightarrow O^* + H_2$ | 0.41 | -0.26 | 0.33 | -0.24 | 0.02 |
| $\frac{1}{2}H_2 \rightarrow H^*$ (Terrace) | 0 | -0.21 | 0.16 | 0.02 | 0.23 |
| $\frac{1}{2}H_2 \rightarrow H^*$ (Beside step) | 0 | -0.21 | 0.08 | -0.06 | 0.15 |
| $\frac{1}{2}H_2 \rightarrow H^*$ (Near step) | 0 | -0.21 | 0.15 | 0.01 | 0.22 |
| $H_2$ | 0.41 | | 0.27 | | |
| H* (Terrace) | 0 | | 0.16 | | |
| H* (Beside step) | 0 | | 0.08 | | |
| H* (Near step) | 0 | | 0.15 | | |
| O* | 0 | | 0.06 | | |
| OH* | 0 | | 0.32 | | |

adsorption free energies to then calculate the free energy as a function of applied potential and pH as follows:

$$\Delta G_{\mathrm{O}^*}(\mathrm{pH}, \mathrm{U_{SHE}}) = \Delta G^0_{\mathrm{O}^*} + 2k_BT \ln 10 \ \mathrm{pH} - 2e\mathrm{U_{SHE}} \tag{S6}$$

$$\Delta G_{\mathrm{OH}^*}(\mathrm{pH}, \mathrm{U_{SHE}}) = \Delta G^0_{\mathrm{OH}^*} + k_BT \ln 10 \ \mathrm{pH} - e\mathrm{U_{SHE}} \tag{S7}$$

$$\Delta G_{\mathrm{H}^*}(\mathrm{pH}, \mathrm{U_{SHE}}) = \Delta G^0_{\mathrm{H}^*} - k_BT \ln 10 \ \mathrm{pH} + 2e\mathrm{U_{SHE}} \tag{S8}$$

Taking these calculated values and normalizing by the surface area of their corresponding surface phase, we can then compare values to predict the most thermodynamically stable phase as a function of pH and $\mathrm{U_{SHE}}$.

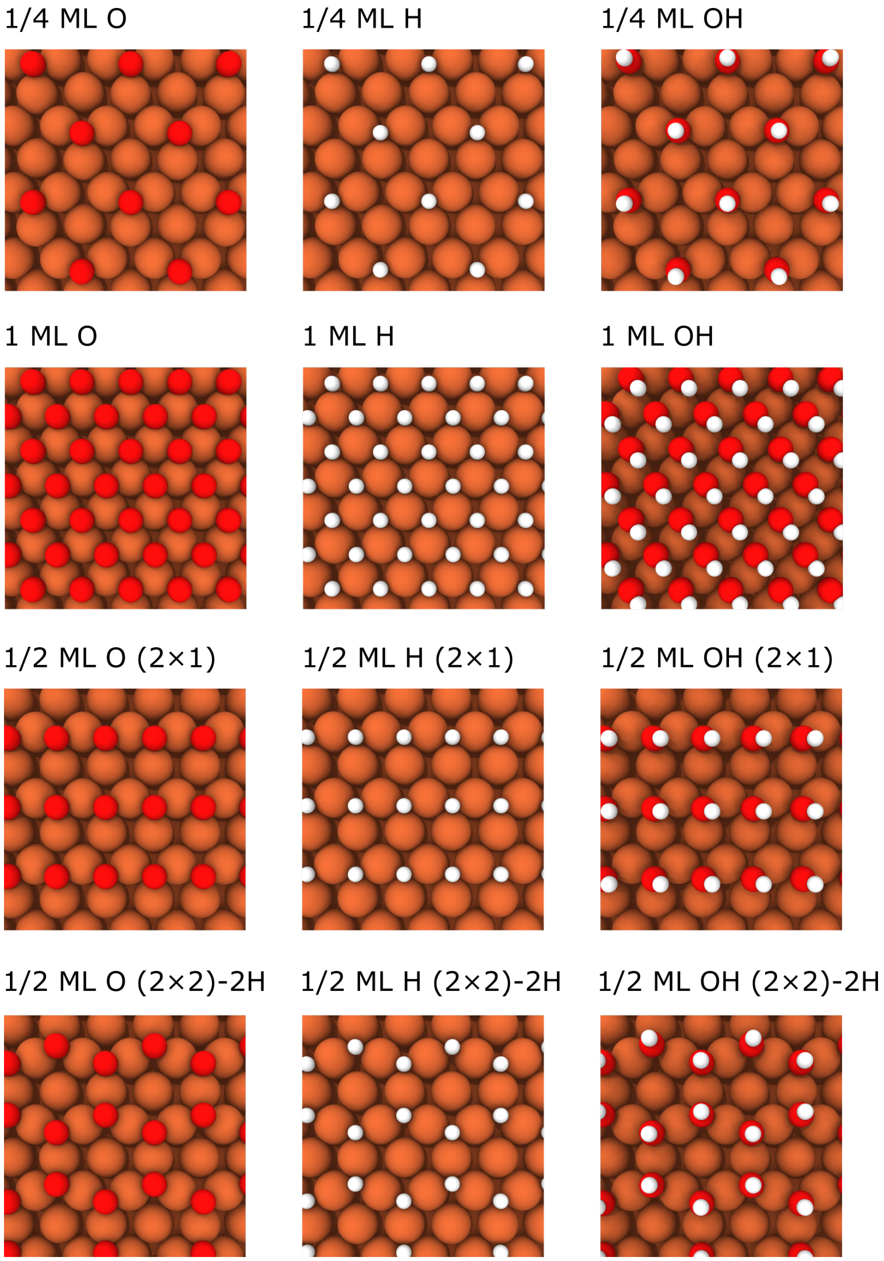


Figure S2: Surface phases for adsorbates at coverages 1/4 ML, 1 ML and 1/2 ML

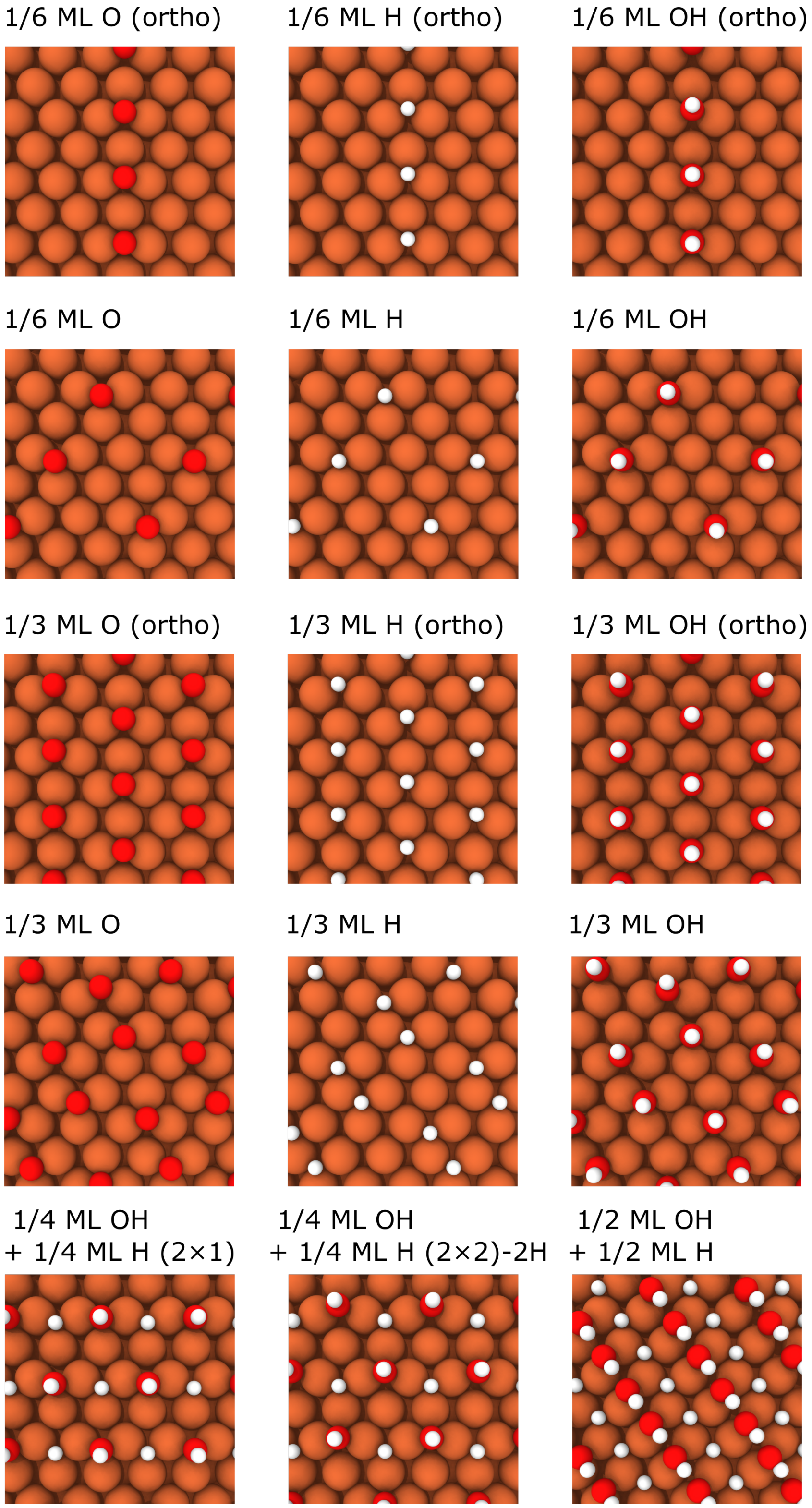


Figure S3: Surface phases for adsorbates at coverages 1/6 ML, 1/3 ML as well as mixed OH + H phases

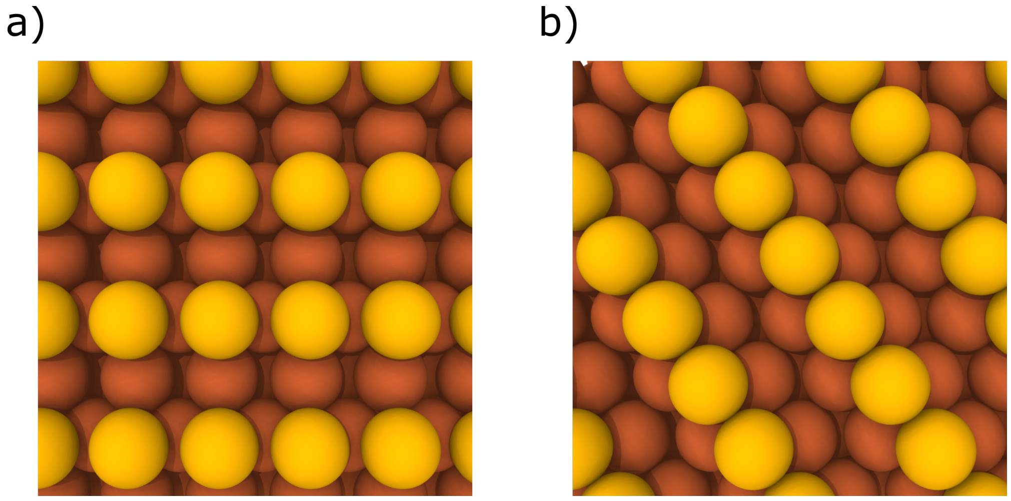


Figure S4: Illustration of two configurations of Fe at 1/2 ML. Yellow atoms indicate the deposited iron atoms. The supercell has been repeated to visualize the differing patterns when considering periodic boundary conditions

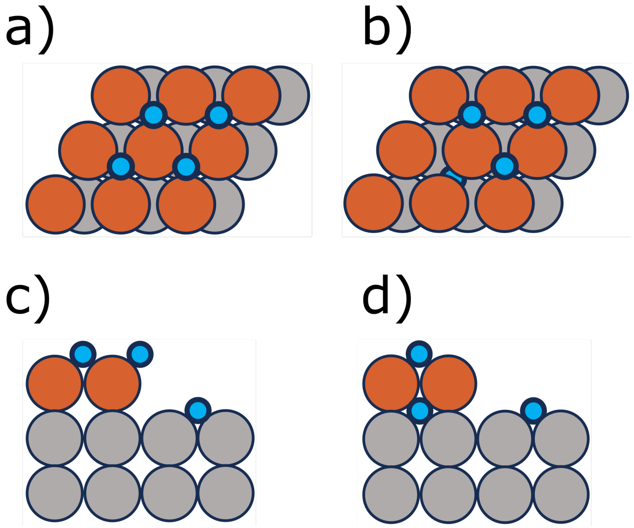


Figure S5: Schematics illustrating absorption structures on hydrogenated surfaces with hydrogen placed a) at terrace surface hollow site (top and side views), b) below terrace short bridge site (top and side views), c) bridge step site, d) below short bridge site near step edge. Outermost surface iron atoms are brown, subsurface iron atoms are grey, and hydrogen atoms are blue.

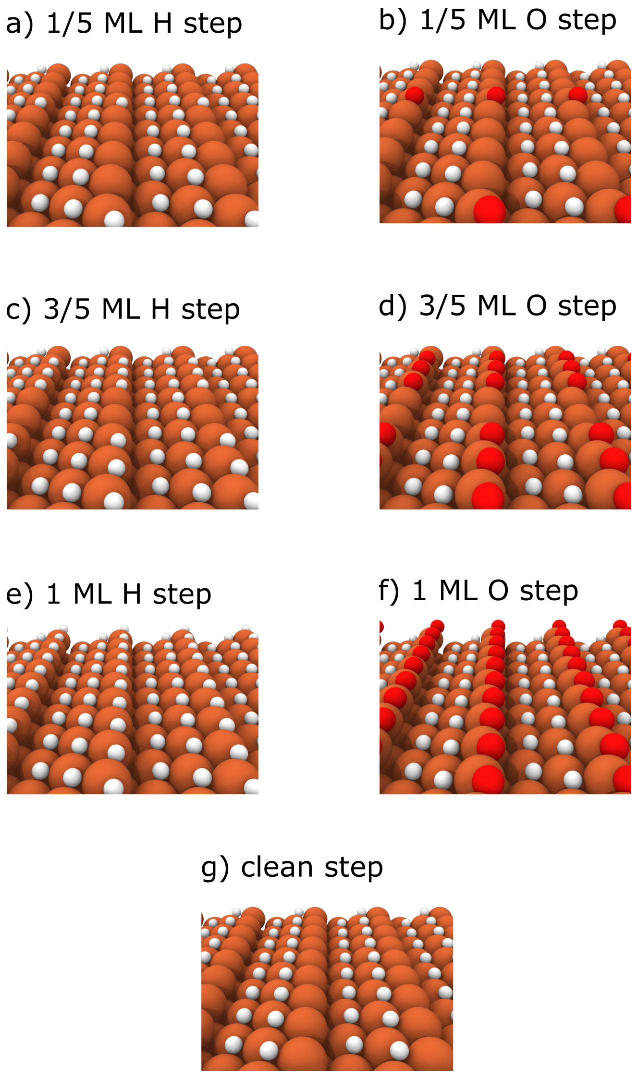


Figure S6: a) Step phase with 1/5 ML H coverage of the step b) Step phase with 1/5 ML O coverage of the step c) Step phase with 3/5 ML H coverage of the step d) Step phase with 3/5 ML O coverage of the step e) Step phase with 1 ML H coverage of the step f) Step phase with 1/5 ML H coverage of the step g) no adsorbates on the step. All surface step phases have 1 ML H coverage on the Fe110 terrace

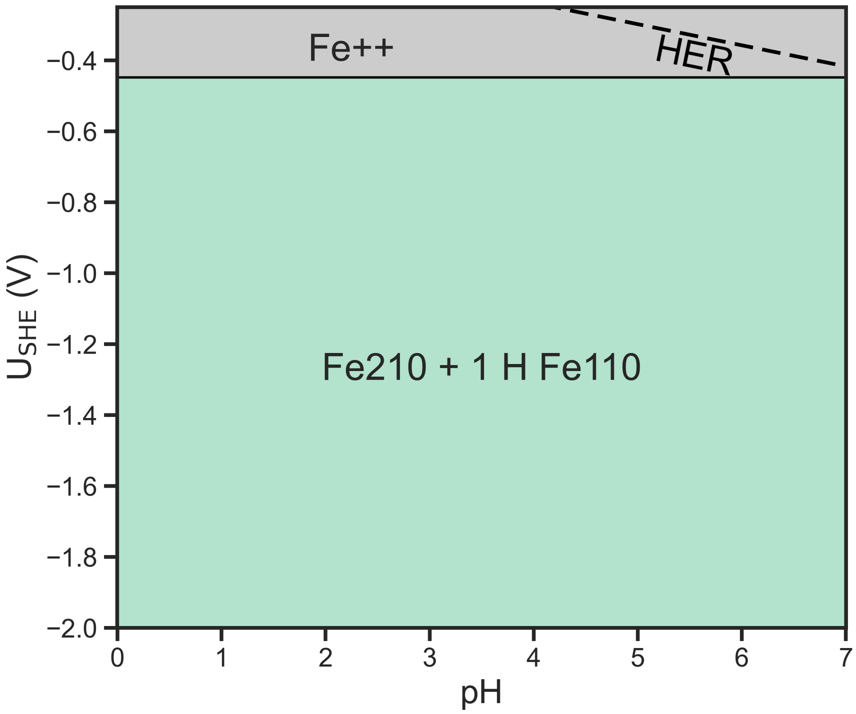


Figure S7: Surface Pourbaix diagram on Fe210 step with fully hydrogenated Fe110

# References

(1) Nørskov, J. K.; Rossmeisl, J.; Logadottir, A.; Lindqvist, L.; Kitchin, J. R.; Bligaard, T.; Jonsson, H. Origin of the overpotential for oxygen reduction at a fuel-cell cathode. The Journal of Physical Chemistry B **2004**, 108, 17886–17892.
